# Fecal and Serum Metabolomic Signatures and Microbial Community Profiling of Postmenopausal Osteoporosis Mice Model

**DOI:** 10.3389/fcimb.2020.535310

**Published:** 2020-11-27

**Authors:** Kaicheng Wen, Lin Tao, Zhengbo Tao, Yan Meng, Siming Zhou, Jianhua Chen, Keda Yang, Wacili Da, Yue Zhu

**Affiliations:** ^1^ Department of Orthopaedics, First Affiliated Hospital of China Medical University, Shenyang, China; ^2^ Department of Orthopaedics, Shengjing Hospital of China Medical University, Shenyang, China

**Keywords:** osteoporosis, gut microbiota, metabolomics, cholic acid, mice

## Abstract

**Background:**

Multiple studies have shown that an imbalance in the intestinal microbiota is related to bone metabolism, but the role of the intestinal microbiota in postmenopausal osteoporosis remains to be elucidated. We explored the effect of the intestinal microbiota on osteoporosis.

**Methods:**

We constructed a postmenopausal osteoporosis mouse model, and Micro CT was used to observe changes in bone structure. Then, we identified the abundance of intestinal microbiota by 16S RNA sequencing and found that the ratio of Firmicutes and Bacteroidetes increased significantly. UHPLC-MS analysis was further used to analyze changes in metabolites in feces and serum.

**Results:**

We identified 53 upregulated and 61 downregulated metabolites in feces and 2 upregulated and 22 downregulated metabolites in serum under OP conditions, and interestedly, one group of bile acids showed significant differences in the OP and control groups. Network analysis also found that these bile acids had a strong relationship with the same family, Eggerthellaceae. Random forest analysis confirmed the effectiveness of the serum and fecal models in distinguishing the OP group from the control group.

**Conclusions:**

These results indicated that changes in the gut microbiota and metabolites in feces and serum were responsible for the occurrence and development of postmenopausal osteoporosis. The gut microbiota is a vital inducer of osteoporosis and could regulate the pathogenesis process through the “microbiota-gut-metabolite-bone” axis, and some components of this axis are potential biomarkers, providing a new entry point for the future study on the pathogenesis of postmenopausal osteoporosis.

## Introduction

Postmenopausal osteoporosis (OP), the most common type of primary osteoporosis, poses a large threat to the health of women around the world (Watts et al., 2010; Pagnotti et al., 2019). OP progresses rapidly and refers to ovarian function decline due to a decrease in oestrogen levels, which leads to more osteoclast absorption than osteoblast-mediated bone formation, characterized by a decrease in the amount of bone tissue per unit volume and by the microarchitectural deterioration of bone tissue (Eastell et al., 2016). A decrease bone density can lead to an increased risk of bone fragility fractures, resulting in pain, disability, and loss of functional independence (Forsén et al., 1999; Orwig et al., 2006). However, drugs based on our current understanding of osteoporosis pathogenesis cannot completely prevent the occurrence and development of postmenopausal osteoporosis (Jian and Sambrook, 2011; Black and Rosen, 2016a).

The gut microbiota has been shown to interact with various organs and systems in the body (Feng et al., 2018), representing an important influencing factor of metabolic health (Ke et al., 2019). The development of new high-throughput sequencing technologies has facilitated the large-scale analysis of the metabolic characteristics of intestinal microbial communities and provided the possibility of a new therapeutic intervention approach, especially for various metabolic diseases in recent years (Zhang et al., 2017; Liu et al., 2017; Meng et al., 2018). Wang found that the gut microbiota regulates obesity through NFIL3, and the circadian clock and Akkermansia could control islet autoimmunity to influence the severity of type 1 diabetes (Wang et al., 2017). It was also reported that high fibre intake led to changes in the gut microbiota and prevented the development of hypertension (Marques et al., 2016). Postmenopausal osteoporosis is a systemic metabolic disease (Black and Rosen, 2016b), and the relationship with gut microbiota remains to be further explored. Increasing evidence has shown that gut microbiota can affect bone formation. Li et al. found that germ-free mice had higher trabecular density than mice fed regular feed. However, when the intestinal microbiota was recolonized, both trabecular density and cortical cross-sectional area were decreased, indicating that the intestinal microbiota is closely related to bone metabolism (Li et al., 2016). The intestinal microbiota may affect bone metabolism through the immune system, endocrine system, or ion absorption (Gilman and Cashman, 2006; Sjogren et al., 2012; Yan et al., 2016), and either pathway is closely related to the concentration of various metabolites in the blood and intestines. The gut microbiota exerts biological effects by producing specific metabolites that act on the intestinal wall or enter the blood to regulate target organs (Rooks and Garrett, 2016; Dalile et al., 2019). However, the bacteria, metabolites, and mechanisms by which intestinal microbiota affect postmenopausal osteoporosis are still unknown.

Therefore, we studied the effect of the intestinal microbiota on the fecal and serum metabolism phenotype under the condition of postmenopausal osteoporosis. We also applied an integrated approach of 16S rRNA gene sequencing combined with blood and fecal ultra-high-performance liquid chromatography-mass spectrometry (UHPLC-MS) analysis to determine whether specific bacterial genera and metabolites were associated with postmenopausal osteoporosis. Random forest (RF) analysis of this model was performed to verify the effectiveness of the model. This study first evaluated the functional metabolic interactions between the microbial species in postmenopausal osteoporosis, resulting in a new breakthrough in the study of the pathogenesis of postmenopausal osteoporosis.

## Materials and Methods

### Animal

We purchased fourteen 10-week-old C57BL6/J female mice from the Laboratory Animal Department of China Medical University. They were raised without specific pathogens. The mice were fed sterile food and autoclaved water ad libitum under a 12 h light cycle. After feeding for one week under this condition, we randomly divided the mice into a postmenopausal osteoporosis group and a control group, with seven mice in each group, and performed ovariectomy on the mice in the OP group to construct postmenopausal osteoporosis models. After successfully establishing the model, we continued to raise the mice for another 10 weeks under the above conditions and then collected femoral samples from each group of mice and removed excess tissue. Next, we fixed the femoral sample with 4% paraformaldehyde for 48 h, performed a microcomputer tomography (Micro CT) examination and evaluated the results. All animal operations in our experiments were performed in strict accordance with the National Institutes of Health (NIH) Guidelines for the Care and Use of Experimental Animals and were approved by China Medical Univeristy Institutional Animal Care and Use Committee (Shenyang, China)

### Micro CT

We used Micro CT (SkyScan 1276, Bruker) to analyze 100 sections from the growth plate of each femur to observe the differences in the volume and structure of cortical and trabecular bone between the two groups. We obtained the following parameters of the sample through built-in software (NRecon, DataViewer CTAn version: 1.17.7.2): trabecular volume percentage (BV/TV), cortical volume (Ct. V), cortical thickness (Ct. Th), trabecular number (Tb. N), trabecular space (Tb. Sp), and trabecular thickness (Tb.Th).

### Feces and Serum Collection

After raising the mice for 70 days, we collected at least two fecal pellets from each mouse, one for metabolic analysis and one for microbial analysis. Immediately after collecting the fecal samples, we placed them in a sterile centrifuge tube and stored them at -80°C for further analysis. Serum samples were collected in the last step of this study. The mice were anaesthetized with isoflurane, and blood was collected. The blood was centrifuged to separate the serum, which was also stored frozen at -80°C for the following metabolic analysis.

### 16S rRNA Microbial Community Analysis

We used the CTAB/SDS method to extract total DNA from stool samples. Analysis was conducted at Novogene Co., Ltd. (Beijing, China). Based on previously reported studies, we selected the V3-V4 region of the 16S rRNA gene using custom barcode universal bacterial primers 338F (5’-barcode-ACTCCTACGGGA,GGCAGCA-3’) and 806R (5’-GGACTACHVGGGTWTCTCATAT-3’) (Chen et al., 2019).

Same volume of 1×loading buffer (contained SYB green) with PCR products and operate electrophoresis on 2% agarose gel for detection were mixed together. PCR products was mixed in equidensity ratios. Then, mixture PCR products was purified with GeneJETTM Gel Extraction Kit (Thermo Scientific). Sequencing libraries were generated using Ion Plus Fragment Library Kit 48 rxns (Thermo Scientific) following manufacturer’s recommendations. The library quality was assessed on the Qubit@ 2.0 Fluorometer (Thermo Scientific). At last, the library was sequenced on an Ion S5TM XL platform and 400/600 bp single-end reads were generated.

Next, based on the threshold of 97% sequence similarity, we used Usearch to cluster the filtered sequences into operational classification units (OTUs) and classify them according to the Greengenes Database. The products were analyzed using the QIIME software package (Version 1.9.1) (Caporaso et al., 2010). Finally, we used the vegan package in R (version 3.2.1) to perform a Bray-Curtis differential analysis of intestinal flora changes.

### Feces and Serum Metabolomic Analysis Preparation

We thawed the fecal samples on ice, all samples were done individually not pooled. We added 100 mg feces from each sample to precooled 50% methanol and mixed thoroughly by vortexing. The samples were then incubated on ice for 5 min and centrifuged (15,000 x g) at 4°C for 15 min, and the supernatant was retained. The supernatant was stored at -80°C until subsequent LC-MS analysis.

After thawing the serum samples on ice, we added 20 μl of serum from each sample to 80 μl of precooled formaldehyde containing internal standard. The mixture was vortexed for 60 seconds to mix thoroughly. Then, the mixture was incubated at -20°C for 12 h and centrifuged (4°C, 12,000 rpm, 15 min). The supernatant was collected and dried under a stream of nitrogen. The dried extracts were resuspended for subsequent LC-MS analysis. A quality control (QC) sample was obtained by mixing an equal quantity of all samples.

### UHPLC-MS/MS Analysis

We used a Vanquish UHPLC system (Thermo Fisher, 100 × 2.1 mm, 1.9 μm) to perform chromatographic separation of the samples at a constant temperature of 40°C and an Orbitrap Q Exactive series mass spectrometer (Thermo Fisher) to detect eluted metabolites. Specific type of column used in the UHPLC-MS/MS analysis was C18. The sample injection volume was 5 μl, and the column flow rate was maintained at 0.2 mL/min. The mobile phase contained two solvent eluents. In positive mode, eluent A was 0.1% FA in water, and eluent B was methanol; in negative mode, eluent A was 5 mM ammonium acetate with a pH of 9.0, and eluent B was methanol. The gradient elution was 2% B for 1.5 min, 2-100% B for 12.0 min, 100% B for 14.0 min, 100-2% B for 14.1 min, and 2% B for 17 min. To analyze the samples, we set the mass spectrometer spray voltage to 3.2 kV, the capillary temperature to 320°C, the sheath gas flow rate to 35 arb, and the auxiliary gas flow rate to 10 arb.

### Database Search

We used Compound Discoverer 3.1 (CD3.1, Thermo Fisher) to process the data generated by UHPLC-MS/MS. We used the following parameters: retention time tolerance, 0.2 min; actual mass tolerance, 5 ppm; signal strength tolerance, 30%; signal-to-noise ratio, 3; and minimum intensity, 100,000. Then, we compared the peaks of each metabolite and observed the differences. We, next, normalized the peak intensities to predict the molecular formula of the metabolites. To analyze the peaks qualitatively and quantitatively, we matched the peaks with the mzCloud (https://www.mzcloud.org/), mzVault and MassList databases. Finally, we performed statistical analysis on the data using statistical software (R, version 3.4.3; Python, version 2.7.6; CentOS, version 6.6). If the data were not normally distributed, normal transformations were attempted using the area normalization method. These metabolites were annotated using the KEGG database (http://www.genome.jp/kegg/), HMDB database (http://www.hmdb.ca/) and Lipidmaps database (http://www.lipidmaps.org/). Principal co-ordinate analysis (PCoA) and orthogonal partial least squares discriminant analysis (OPLS-DA) were performed with metaX. A t-test was used to calculate the P-value. If VIP >1, P-value <0.05, and FC ≥2 or FC ≤0.5, the differences between the metabolites were significant. We plotted the results on a volcano map. We set the abscissa of the volcano chart to log2 (fold change) and the ordinate to -log10 (P-value) to determine the changes in the metabolites. For clustering heat maps, the data were normalized using z-scores of the intensity areas of differential metabolites and were plotted by the Pheatmap package in R language. The correlations between differential metabolites were analyzed by R language. R language was used to analyze whether there was a correlation between differential metabolites. If the correlation was statistically significant, correlation plots were plotted by the corrplot package in R language. The functions of these metabolites and metabolic pathways were studied using the KEGG database. Metabolic pathway enrichment analysis of differential metabolites was performed. When the ratio was satisfied by x/n > y/N, the metabolic pathways were considered enriched, and when the P-value of metabolic pathway <0.05, metabolic pathways were considered significantly enriched.

## Results

### Evaluation of Femoral Bone Structure

**Figure 1:**
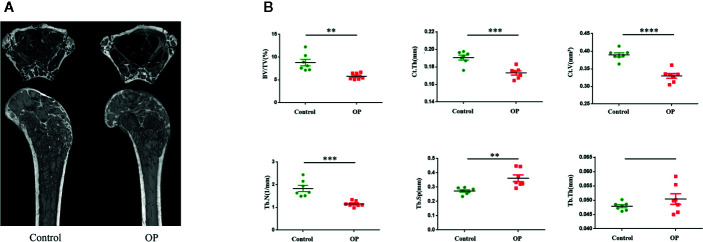
**(A)** Images of representative 3-dimensional Micro CT reconstructions of examined femurs from each group. **(B)** Evaluation of femoral bone of control and OP mice by Micro CT after 70 days. Comparision of BV/TV, Ct.Th,Ct.V, Tb.N,Tb.Sp, Tb.Th in the two groups. (n = 7 mice per group in all panels. Data are expressed as mean ± SEM. *P < 0.05, **P < 0.01, ***P < 0.001, and ****P < 0.0001).

The results of Micro CT (**Figure 1A**) showed that the values of BV/TV, Ct. V, Ct. Th, and Tb. N were lower in the OP group than in the control group. However, Tb. Sp of the OP group was higher than that of the control group. There was no significant difference in Tb. Th between the two groups (**Figure 1B**).

### Gut Microbiota Significantly Distinguish the OP Group From the Control Group

**Figure 2:**
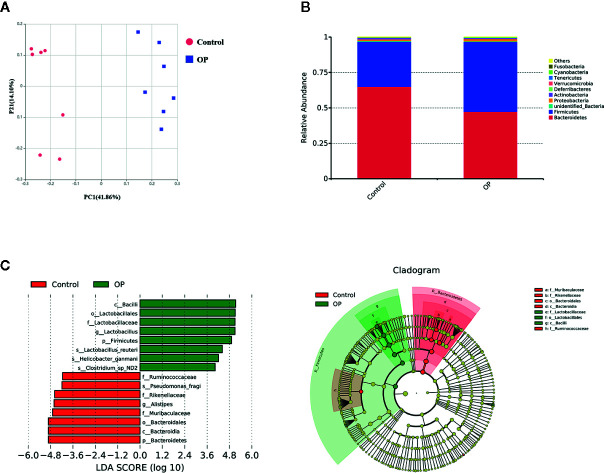
**(A)** PCoA analysis of the gut microbiota. PC1 = 41.86%, PC2 = 14.10% OP group: red; OP group: blue, Control group: red, n = 7. **(B)** Difference of gut microbiota between OP group and control group at phylum level, n = 7. **(C)** LEfSe analysis of gut microbiota for OP group: green, Control group: red, n = 7, LDA score >4.0. Red represents increased flora in OP mice; Green represents increased flora in control mice.

The OTU analysis results revealed the microbiota changes in the OP group. A total of 802 OTUs were generated from OP and normal mice (n = 7), including 21 phyla, 30 classes, 61 orders, 108 families, 180 genera, and 132 species. We used principal coordinate analysis (PCoA) to observe the difference between the microbiota in the OP group and the control group (**Figure 2A**). Each point in the graph represents a single sample. The figure shows that the flora in the OP group and the control group are clearly divided into two regions, indicating that the flora in the two groups was significantly different. After that, we performed a phylum-level analysis, as shown in **Figure 2B**. The amount of Firmicutes and Bacteroidetes and the percentage of Proteobacteria were significantly increased, while Verrucomicrobia was significantly reduced in the OP group compared with the control group. Next, we performed an LDA effect size analysis (LEfSe) with LDA fold = 4, and the relationship between different microbiota from the phylum level to the genus level is shown in the cladogram (**Figure 2C**). As shown in the LDA score map, in the OP mice, c_Bacilli, o_Lactobacillales, f_Lactobacillaceae, g_Lactobacillus, p_Firmicutes, s_Lactobacillus_reuteri, s_Helicobacter_ganmani, and s_Clostridium_sp_ND2 were significantly increased, while in the control mice, f_Ruminococcaceae, s_Pseudomonas_fragi, f_Rikenellaceae, g_Alistipes, f_Muribaculaceae, o_Bacteroidales, c_Bacteroidia, and p_Bacteroidetes were significantly increased.

### Metabolomics Analysis of Fecal Samples From Postmenopausal Osteoporosis Mice

**Figure 3:**
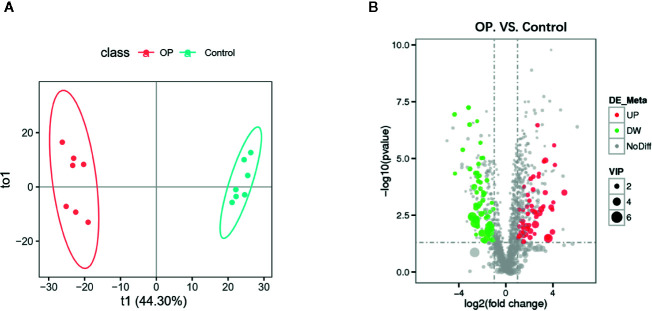
**(A)** OPLS-DA scores plot of fecal metabolite profiling between the OP and control groups. **(B)** Volcano plot analysis of fecal metabolites (VIP > 1, |P(corr)| ≥ 0.5, jackknifed 95% confidence intervals).

We performed OPLS-DA analysis on the data obtained by LC-MS (**Figure 3A**) to identify the differences in fecal metabolites between the two groups. The metabolite profiles of the two groups showed good separation, indicating that OP can cause changes in biomarkers in feces. OPLS-DA classification modeling, the sample is divided into training set and test set at a ratio of 6:1, using 7-fold cross-validation, each time one copy is selected as the test set test sample, and the remaining 6 copies are used as the training set Training modeling. R^2^ and Q^2^ were showed in **Figure S1**. After the analysis, we obtained a total of 114 metabolites with changes in the OP model by P-value (Hagan et al., 2019), of which 53 were upregulated and 61 were downregulated (**Figure 3B**). We mapped volcano plots for the 1,255 fecal metabolites identified by LC-MS. Among these, 42 metabolites were identified as potential biomarkers based on the HMDB database. We listed the Log2FC, P-value and VIP values of the metabolites in **Table 1**. Compared with Control group the levels of 4-Phenylbutyric acid, b-Pseudouridine, Uridine, Adenosine, Biotin were increased and Riboflavin, Ritalinic acid, Glycyl-L-leucine, Gly-Val, Gly-Tyr were decreased.

**Table 1:** Differential fecal metabolites.

Name	log2FC	Pvalue	VIP	Up Down
**4-Phenylbutyric acid**	2.520114	0.008154	3.292082	up
**Riboflavin**	-1.54385	0.003643	1.584122	down
**b-Pseudouridine**	2.068796	0.029984	1.766358	up
**Uridine**	1.508218	0.045368	1.730744	up
**Adenosine**	1.702021	0.022402	1.98056	up
**Biotin**	1.469365	0.00183	1.142501	up
**Ritalinic acid**	-1.7891	0.044506	1.377868	down
**2-Hydroxyphenylalanine**	2.710969	3.49E-07	1.323967	up
**Glycyl-L-leucine**	-3.06621	2.87E-05	1.742255	down
**Gly-Val**	-2.58124	4.49E-05	1.735141	down
**Gly-Tyr**	-2.16333	0.000113	1.346554	down
**L-threo-3-Phenylserine**	-1.82545	0.000352	1.384437	down
**Ala-Ile**	-2.2229	0.000994	2.02748	down
**N-Acetyl-L-leucine**	-2.10276	0.002756	2.228996	down
**Leucylproline**	-1.31182	0.019222	3.245156	down
**gamma-Glutamyltyrosine**	-2.4517	2.30E-07	1.017503	down
**L-(-)-Methionine**	-1.93278	0.000311	1.343952	down
**Methionine sulfoxide**	-1.06576	0.001708	1.28186	down
**Ornithine**	-1.1902	0.009353	1.792292	down
**L-Tyrosine**	-2.17247	0.000215	1.533334	down
**Valine**	-2.34421	0.000101	1.522017	down
**Catechol**	1.478887	0.017643	1.387654	up
**Hyodeoxycholic Acid**	4.121678	2.67E-06	1.20456	up
**Glycocholic acidGlycocholic acid**	-3.6759	4.17E-06	1.428059	down
**Stercobilin**	4.990742	0.000317	2.467178	up
**D-Glucose 6-phosphate**	3.965526	0.001797	1.234257	up
**Deoxyribose 5-Phosphate**	1.925479	0.010653	1.030104	up
**4-Hydroxybenzaldehyde**	-1.61006	9.24E-05	1.451096	down
**Ergosterol peroxide**	1.135775	0.001783	1.197758	up
**Isopropyl myristate**	2.32473	0.000235	1.571395	up
**Sildenafil-d3**	-2.49217	0.00031	1.300368	down
**2-Hydroxy-4-methylthiobutanoic acid**	-2.55016	0.004946	3.924234	down
**2-Hydroxycaproic acid**	-2.61195	0.007083	4.482585	down
**Docosanamide**	-2.69821	0.00018	2.402933	down
**2-Hydroxycinnamic acid**	-2.24045	5.77E-05	1.378673	down
**Skatole**	-3.09426	3.25E-07	1.556727	down
**DL-Tryptophan**	-3.18289	5.82E-08	1.432171	down
**Indole-3-lactic acid**	-1.95456	0.000697	1.641163	down
**Isohomovanillic acid**	-1.56827	0.018266	1.886425	down
**7-Methylguanine**	-1.04554	0.03577	2.476467	down
**Guanine**	-1.60688	0.031909	3.136626	down
**Cytosine**	-1.56665	0.013968	2.442863	down

### Metabolomics Analysis of Serum From Postmenopausal Osteoporosis Mice

**Figure 4:**
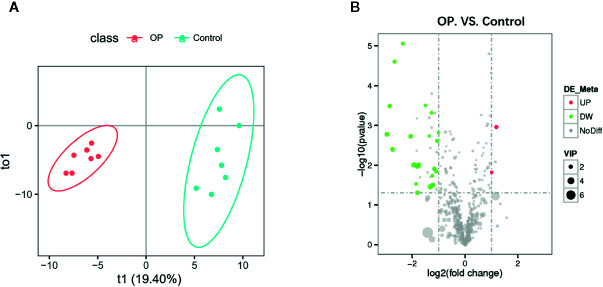
**(A)** OPLS-DA scores plot of serum metabolite profiling between the OP and control groups. **(B)** Volcano plot analysis of serum metabolites (VIP > 1, |P(corr)| ≥ 0.5, jackknifed 95% confidence intervals).

We performed OPLS-DA analysis on the data obtained by LC-MS (**Figure 4A**) to identify the differences in serum metabolites between the two groups. R^2^ and Q^2^ were showed in **Figure S2**. The metabolite profiles of the two groups showed good separation, indicating that OP can cause changes in biomarkers in serum. We mapped volcano plots for the 515 serum metabolites identified by LC-MS. After the analysis, we obtained a total of 24 metabolites with changes in the OP model by P-value, of which 2 were upregulated and 22 were downregulated (**Figure 4B**). Among these, 16 metabolites were considered potential biomarkers of OP. We listed the Log2FC, P-value and VIP values of the metabolites in **Table 2**. Compared with Control group the levels of Tetrahydro-11-deoxycortisol, 4-Methylphenol, 6-Phosphogluconic acid, Deoxycholic acid, 5-Hydroxytryptophol were decreased and 2-Hydroxymyristic acid were increased.

**Table 2:** Differential serum metabolites.

Name	log2FC	P-value	VIP	Up Down
**Tetrahydro-11-deoxycortisol**	-2.35364	8.72E-06	1.465754	down
**4-Methylphenol**	-2.85508	0.000329	1.773945	down
**6-Phosphogluconic acid**	-1.26675	0.000481	1.357323	down
**2-Hydroxymyristic acid**	1.188196	0.001099	1.736772	up
**Deoxycholic acid**	-2.96145	0.00168	1.997309	down
**5-Hydroxytryptophol**	-2.05355	0.001885	2.036391	down
**D-Mannose 6-phosphate**	-1.05951	0.002457	1.616874	down
**7-Ketodeoxycholic acid**	-1.73967	0.009479	1.178995	down
**D-Ribulose 5-phosphate**	-1.80248	0.010638	2.468253	down
**N-Phenylacetylglycine**	-1.16033	0.012151	1.582213	down
**Acetylcarnitine**	-1.09344	0.014461	1.522388	down
**Hydrocinnamic acid**	-1.23681	0.018479	1.425951	down
**Progesterone**	-1.85365	0.029654	1.175589	down
**Cholic acid**	-1.21818	0.031485	2.146499	down

### Correlations of Gut Microbial Genera with the Metabolome of Postmenopausal Osteoporosis Mice

**Figure 5:**
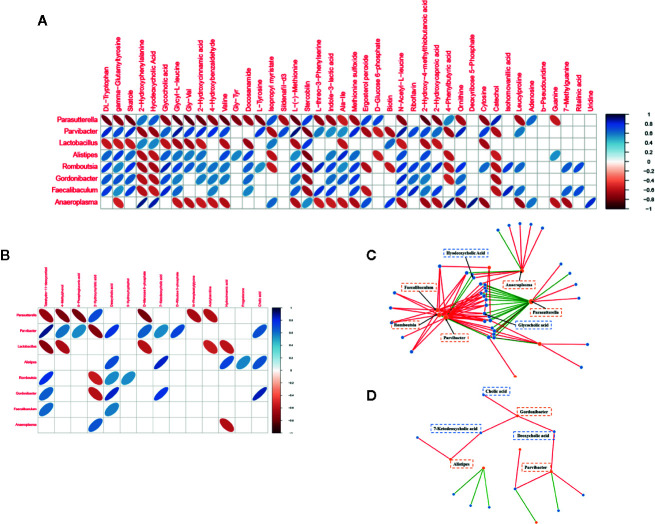
Relationships of gut microbial genera with metabolites in OP mice. **(A)** Heat map to display the correlations of gut microbial genera and fecal metabolites. **(B)** Heat map to display the correlations of gut microbial genera and serum metabolites. **(C)** Network analysis between well-predicted bacteria and fecal metabolites. **(D)** Network analysis between well-predicted bacteria and serum metabolites.

We plotted a heat map to display the correlations of gut microbial genera and differential metabolites (**Figures 5A, B**). The relationships between well-predicted bacteria and fecal and serum metabolites are plotted in **Figure 5C** (|r| > 0.7) and **Figure 5D** (|r| > 0.7), respectively. There were multiple correlations between the gut microbiota at the genus level and metabolites, especially bile acids. For example, according to the fecal metabolomic and 16S analyses, hydrodeoxycholic acid was positively correlated with Anaeroplasma (r = 0.862) and Parvibacter (r = 0.700). Glycocholic acid was positively correlated with Faecalibaculum (r = 0.821), Romboutsia (r = 0.856), and Gordonibacter (r=0.707) and a negative correlation with Parasutterella (r = -0.728). Serum deoxycholic acid had positive relationships with Parvibacter (r = 0.775) and Gordonibacter (r = 0.831), and Gordonibacter also had positive correlations with two other cholic acids, 7-ketodeoxycholic acid (r = 0.789) and cholic acid (r = 0.854). Gordonibacter and Parvibacter belong to the same family, Eggerthellaceae.

The above results may suggest that the occurrence and development of osteoporosis are closely related to cholic acid metabolism and that the Eggerthellaceae family plays a vital role in this process.

### Random Forest Analysis

**Figure 6:**
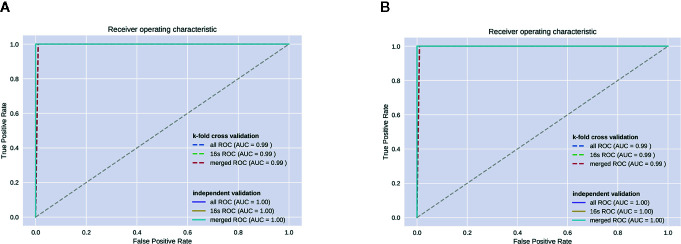
**(A)** Fecal metabolomic and 16S ROC. **(B)** Serum metabolomic and 16S ROC.

We used genus-level RF analysis to test the effectiveness of distinguishing the OP model from the control group (**Figures 6A, B**). The sample is divided into a training set and a test set at a ratio of 4:1, and the average of the false positive rate and true positive rate of 5-fold cross-validation is used to draw ROC curve. Since the independent test set is used for testing, when the AUC of the test set is not significantly reduced relative to the training set, it is considered that there is no overfitting. We obtained an AUC of 0.99 from both the 16S ROC curve and all ROCs.

## Discussion

Beneficial and pathogenic bacteria maintain a balanced state (Icaza-Chávez, 2013). The intestinal microbiota can act as a barrier to prevent invasion by disease-causing microorganisms and influence endocrine organs, such as by providing short-chain fatty acids and vitamins to the host (Sudo and Microbiome, 2014; Neuman et al., 2015). When the balance is disrupted, the gut microbiota will induce pathological processes in their host to cause a variety of severe metabolic diseases (Icaza-Chávez, 2013). Researchers have indicated that intestinal microbiota imbalance might cause bone metabolic disorders (Britton et al., 2014; Ohlsson et al., 2014; Parvaneh et al., 2015). However, the relationship between intestinal microorganisms and host metabolites in osteoporosis has seldom been studied thus far. Through the application of a multiomics correlation network approach, we analyzed the relationships between changes in the microbiota and in the host and identified the association of specific metabolites with the occurrence and development of postmenopausal osteoporosis in this study.

In the 16S rRNA gene sequencing analysis, we observed an increased Firmicutes/Bacteroidetes ratio, and Akkermansia was shown to decrease in our research. These factors all relate to the overactivation of self-immunity. For example, Ansaldo et al. found that Akkermansia induced intestinal adaptive immune responses (Ansaldo et al., 2019) under homeostasis conditions. The ratio of Firmicutes/Bacteroidetes is closely related to circulating short-chain fatty acids (SCFAs) and can shape the immunological environment (Trompette et al., 2014; Marques et al., 2017). Overactivation of self-immunity is a widely accepted osteoporosis pathogenesis (Zupan et al., 2013; Crotti et al., 2015), indicating that this activation may be induced by gut microbiota imbalance.

To clarify how the intestinal flora regulates bone metabolism, we further explored the metabolism of postmenopausal osteoporosis to determine the pathogenic mechanism of OP. Metabolomics is emerging as a tool to discover biomarkers and unravel pathological processes. Lei, using functional metabolomics, found that N-acetylneuraminic acid played a vital role in coronary artery diseases (Black and Rosen, 2016a). Yachida performed fecal metagenomic and metabolomic studies on 616 patients and found that branched-chain amino acids and phenylalanine were very important in the aetiology and diagnosis of intramucosal carcinomas (Yachida et al., 2019). In this research, fecal metabolomics revealed a series of meaningful metabolite changes, such as riboflavin (vitamin B2), which could affect osteoblast differentiation (Chaves Neto et al., 2010). Additionally, a population-based cohort that included 5,053 individuals further showed that dietary riboflavin intake positively influences BMD (Rejnmark et al., 2008). We also found changes in 14 amino acids, one of the important energy sources during bone remodelling that affect bone resident cells through neuronal and hormonal mechanisms (Rendina-Ruedy and Rosen, 2020). To further understand the crosstalk between metabolites and microbiota, correlation analysis was conducted. Gordonibacter and Parvibacter seemed to play a vital role, and they belong to the same family, Eggerthellaceae; moreover, both correlated with bile acid. Hydrodeoxycholic acid, glycocholic acid, deoxycholic acid, 7-ketodeoxycholic acid, and cholic acid all had strong correlations (|r| > 0.7) with these genera.

Microbiota and bile acids have multiple interactions. The microbiota can regulate bile acid synthesis through the bile acid receptors FXR and TGR5, which participate in bile acid metabolism (Sayin et al., 2013). In return, bile acids can modify microbiota abundance by promoting bile-metabolizing bacteria proliferation and inhibit bile-sensitive bacteria proliferation, thereby regulating gut microbiota (Parséus et al., 2016).

**Figure 7:**
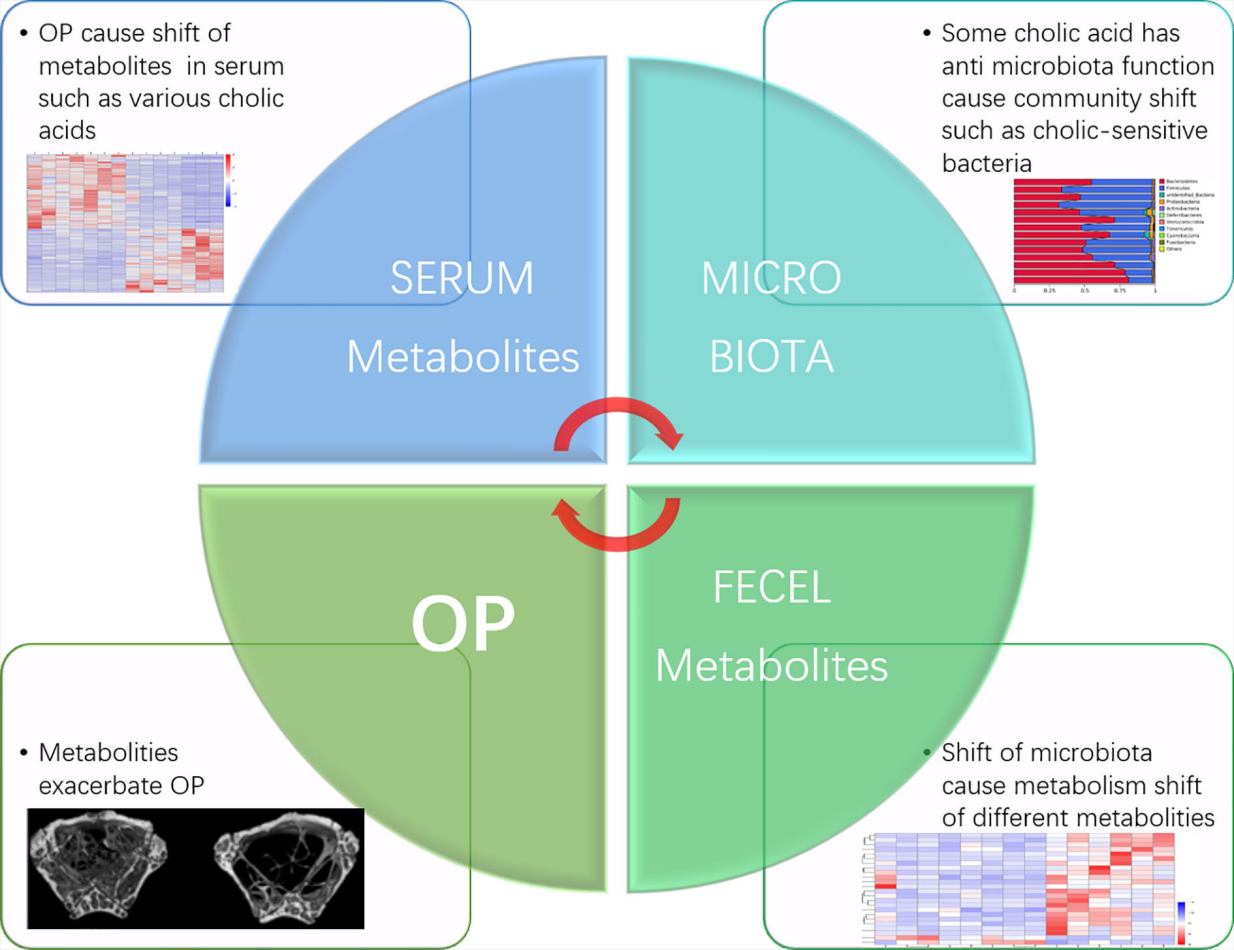
Inferences about intestinal microbiota and metabolites regulate osteoporosis.

Recent research has shown that osteoporosis is commonly seen among patients with chronic cholestasis (Guañabens and Parés, 2018), and TGR5 knockout strongly induced osteoclast differentiation in an OP mouse model (Li et al., 2019). Bile acids can also regulate bone turnover, but the functions of specific bile acids are still unclear. In our results, deoxycholic acid, which acted as a Tgr5 agonist and is produced in the gut (Jensen et al., 2013), was significantly downregulated in the OP group. Based on this result, we infer that OP causes increased bile acid secretion and that the antimicrobial effect of bile acid on intestinal bacteria causes changes in the species abundance of the intestinal flora. This effect may increase bile acid-metabolising flora and reduce bile acid-sensitive flora, which subsequently affects bile acid intestinal metabolism and increases or decreases certain kinds of serum bile acid concentrations, eventually affecting osteoclast activity and causing bone mass loss. Exactly which type of bile acid affects osteoclasts and whether the Eggerthellaceae family is closely related to bile acid metabolism still need to be proven by further experiments (**Figure 7**).

Finally, we used random forest analysis to build classification algorithm to determine whether our results effectively distinguished the OP model group from the control group. Random forest analysis is commonly used to solve “large p, small n” problems. In addition, overfitting is not a problem because the generalization error of the random forest converges (Matsuki et al., 2016). The AUC of our model was 0.99 for both fecal and serum samples, indicating that the model was appropriate to differentiate between OP and normal mice. Although our research confirmed that the OP and normal groups had significant differences in intestinal flora and metabolites, the differences in the blood metabolome were not as obvious as those in the intestine. This finding may indicate that the intestinal microbiota may have a large effect on the body by indirect effects; some metabolites act on the intestinal wall rather than directly in the blood, such as changing the proportion of intestinal Th17 cells and Treg cells to regulate the immune activation level in the body (Britton et al., 2019; Hang et al., 2019). Correlating metabolomics with more omics analyses may lead to new findings.

However, our study has certain limitations. Our sample size was rather small not only for the random forest test but also for the whole experiment. Larger sample size experiment is need in the future to identify ideal biomarker for postmenopausal osteoporosis. And we only found a correlation between osteoporosis intestinal microbiota and metabolites, but not to prove the causal relationship between them, these also needed further research.

## Conclusion

Our research found that the gut microbiota and metabolic regulation were closely related to the occurrence and development of osteoporosis. The gut microbiota is a vital inducer of osteoporosis and could regulate pathogenesis through the “microbiota-gut-metabolite-bone” axis, and some of the components of this axis are potential biomarkers, providing a new entry point for future studies on the pathogenesis and treatment of postmenopausal osteoporosis.

## Data Availability Statement

The original contributions presented in the study are publicly available. This data can be found here: https://www.ncbi.nlm.nih.gov/PRJNA607414.

## Ethics Statement

The animal study was reviewed and approved by Laboratory Animal Department of China Medical University.

## Author Contributions

Conceptualization, KW and LT; Data curation, KW; Formal analysis, WD; Funding acquisition, LT; Investigation, YM and ZT; Methodology, KW and YM; Project administration, LT; Resources, LT; Software, SZ; Supervision, WD and JC; Validation, ZT; Visualization, WD and KY; Writing – original draft, KW, YM and ZT; Writing – review & editing, KW, YZ, ZT and LT. KW and LT contributed equally to this work.

## Funding

This study was funding by Construction of Clinical Medical Research Center of Orthopaedics and Sports Rehabilitation Diseases in Liaoning Province (Grant: 2019416030).

## Conflict of Interest

The authors declare that the research was conducted in the absence of any commercial or financial relationships that could be construed as a potential conflict of interest.

## Supplementary Material

Supplementary Figure 1 R^2^ and Q^2^ of fecal metabolomic OPLS-DA.

Supplementary Figure 2 R^2^ and Q^2^ of serum metabolomic OPLS-DA.
