# Supplementary figures and images for: Fecal and Serum Metabolomic Signatures and Microbial Community Profiling of Postmenopausal Osteoporosis Mice Model
Source: Front Cell Infect Microbiol. 2020 Nov 27;10:535310. doi: 10.3389/fcimb.2020.535310 (PMC7728697; doi:10.3389/fcimb.2020.535310)

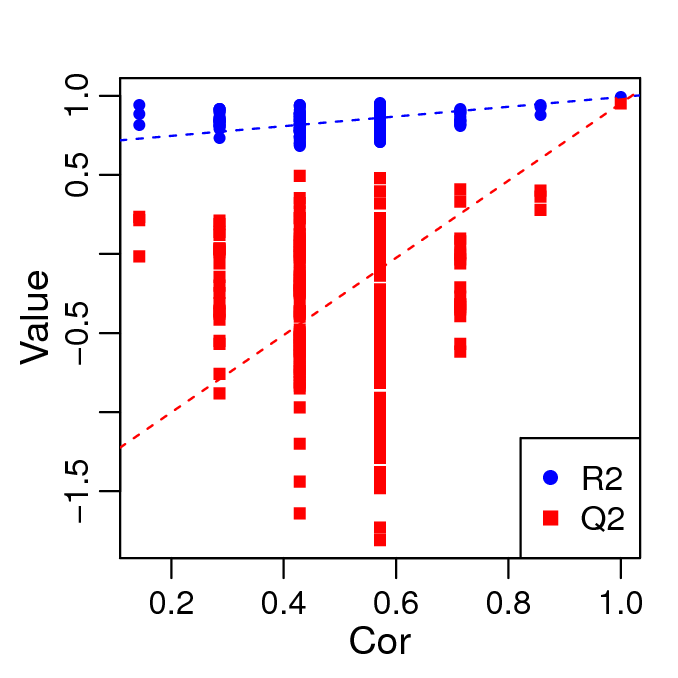

Supplement: Supplementary Figure 1 — R2 and Q2 of fecal metabolomic OPLS-DA. [file Image_1.tif]

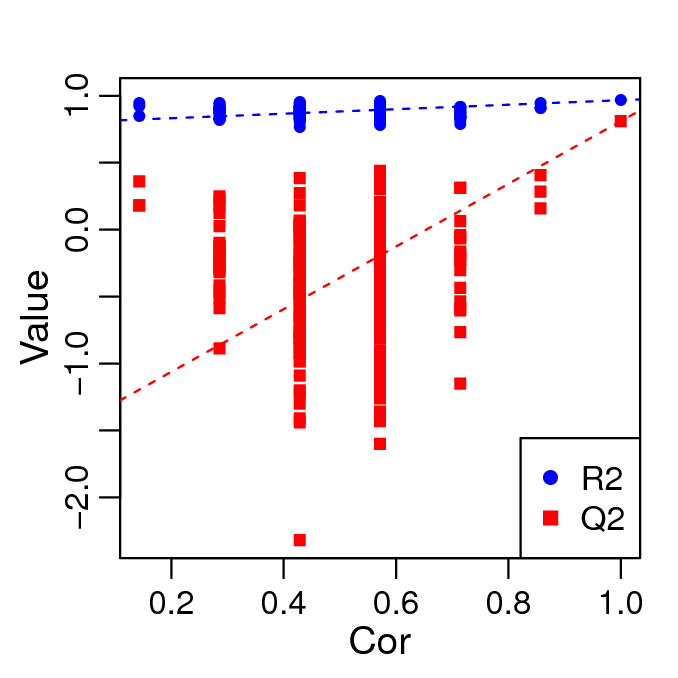

Supplement: Supplementary Figure 2 — R2 and Q2 of serum metabolomic OPLS-DA. [file Image_2.tif]
